# Effectiveness of a self-directed learning program using blended coaching among nursing students in clinical practice: a quasi-experimental research design

**DOI:** 10.1186/s12909-019-1672-1

**Published:** 2019-06-24

**Authors:** Gie-Ok Noh, Dong Hee Kim

**Affiliations:** 1 College of Nursing, Konyang University, 158 Gwanjeodong-ro, Seo-gu, Daejeon, 35365 Korea; 2 College of Nursing, Sungshin University, 76 Ga-gil 55 Dobong-ro, Kangbuk-gu, Seoul 01133 Korea

**Keywords:** Self-directed learning, Coaching, Nursing education

## Abstract

**Background:**

New educational approaches may be necessary to enhance competency in the new generation of students. Self-directed learning and blended coaching have been effective strategies to meet this challenge. However, there has been little research on self-directed learning programs using blended coaching (SDL_BC) in clinical practice. This study aimed to evaluate the effectiveness of a self-directed learning program using blended coaching among nursing students in clinical practice.

**Methods:**

A non-equivalent control group pretest-posttest non-synchronized intervention design was used. The participants were 91 students, comprising an experimental group (*n* = 44) and a control group (*n* = 47). The experimental group was trained using a self-directed learning program with blended coaching. The Self-Directed Learning Competency Questionnaire, Clinical Competency Questionnaire, and Numeric Rating Score for clinical practice satisfaction via a self-report were all used in the assessment. Descriptive statistics, independent t-tests and ANCOVA were used to evaluate self-directed learning competency, clinical competency, and clinical practice satisfaction.

**Results:**

Students in the experimental group showed a significantly higher improvement in competency in the implementation of self-directed learning (F (1,89) = 4.27, *p* = 0.039) and higher satisfaction with clinical practice (t (89) = 3.10, *p* = 0.003) compared with those in the control group.

**Conclusions:**

These results provide evidence that a self-directed learning program using blended coaching is an effective educational approach to improve the implementation part of self-directed learning competency and clinical practice satisfaction among nursing students in clinical practice. Further research is required to investigate whether other variables are associated with clinical competency, competency in planning and evaluation of self-directed learning, or their long-term effects.

## Background

Self-directed learning has become a popular concept in nursing education. The benefits of self-directed learning include independence, professional autonomy, and increased choice and motivation [1]. Many faculty members are attracted to the self-directed learning approach because it enables nursing students to develop independent learning skills as well as accountability, responsibility, and assertiveness, which will be important qualities throughout their careers, allowing them to adapt to the dynamic clinical environment [2, 3]. Therefore, self-directed learning is a necessary and effective strategy for nursing students in their clinical practicum.

Successful integration of the self-directed learning process into existing curricula requires adequate preparation of faculty members and students, particularly at the undergraduate level [2]. Self-directed learning requires a coach to assist and guide students. Effective coaching involves learning from interactions with students, cooperation, exchanging learning materials, and providing encouragement. Coaching has been found to be a good approach to successfully lead students’ self-directed learning [4]. The coach in self-directed learning prepares and motivates students, and helps them set learning objectives, establish learning strategies, manage learning resources, and evaluate their learning results during the self-directed learning process [5]. Therefore, instruction of faculty members in self-directed learning and coaching methods is very important.

Currently, technology-based teaching methods have become mainstream in nursing education [6]. Among them, blended teaching, which combines the strengths of online and offline instruction, is growing rapidly [7]. Online education in clinical practice can provide various advantages, such as facilitating access to training materials or references and communication with instructors. However, online education alone is not enough for clinical practice education. Blended learning integrates online and offline education and can teach skills and help students to overcome their fear of learning and increase their motivation, which have been identified as limitations of online education. In this way, blended coaching can maximize learning outcomes through continuous learning management provided by the teacher, who facilitates and guides the learning process by combining the advantages of online and offline education [8]. Blended coaching is the most important approach to maximize learning effectiveness by appropriately arranging and combining various learning methods according to learning types and characteristics of the online and offline learning environments [9, 10]. Effective coaching provides appropriate guidance, discussion, case studies, and individual and cooperative learning. The blended method also allows for the integration of learning experiences from formal offline training (e.g., on the clinical ward) and online tasks [9]. Finally, student-centered education methods used in blended coaching are effective for self-directed learning. Teachers can easily maintain student motivation and continuous learning through various online and offline methods [8]. For these reasons, blended coaching is the most appropriate way to develop self-directed learning skills. However, there has been little research on self-directed learning programs using blended coaching (SDL_BC) in clinical practice.

Therefore, we aimed to determine the effects of SDL_BC in order to maximize the effectiveness of the clinical practicum, emphasized as self-directed learning competency, clinical competency, and clinical practice satisfaction. The hypothesis that was tested is as follows: students who participated in the SDL_BC will score higher than those in the control group on self-directed learning competency, clinical competency, and clinical practice satisfaction.

## Methods

### Design

This study used a non-equivalent control group design with pre-post non-synchronized intervention.

### Participants and setting

This study targeted junior nursing students from baccalaureate programs at a university located in Seoul, Korea. The participants were chosen by convenience sampling. A total of 92 students agreed to participate, and 91 students were included in the final analysis (one questionnaire had missing items). Data from 47 subjects in the experimental group and 44 subjects in the control group were used. The sample size was calculated using the G*Power 3.1.9 program. The effect size was set to 0.58 based on the results of a previous study that verified the effects of a self-directed learning program for Korean nursing students on self-confidence and satisfaction [11]. For two tail independent t-test between the two groups, the minimum sample sizes were each calculated as 48 for the experimental group and the control group for an effect size of 0.58, significance level of .05, power of .80, and allocation ratio N2/N of 1. The number of samples in this study was fairly small.

### Intervention

We developed the SDL_BC based on Garrison’s self-directed learning model [12, 13], which consists of three dimensions: motivation, self-management, and self-monitoring (Table 1). We designed the program to include motivation by pre-clinical activities, development of self-management skills during practice, and conduction of self-monitoring activities after practice. Each stage consisted of both online and offline activities. Coaching strategies were based on the strategy model of self-regulated learning by Zimmerman & Martinez-Pones [14]. Coaching included not only direct instruction but also feedback about discussions and assignments, review of daily learning objectives and content, and encouragement of activities. All coaching processes prioritized support and interaction with students. The detailed components of the SDL_BC are presented in Table 1. The intervention was implemented by a professor, and students were organized into teams of five to six. During the 2-week intervention, participants performed day and evening shifts.

**Table 1:** Intervention: Self-directed Learning Program using Blended Coaching (SDL_BC)

Self-directed Learning Component/Learning Objectives	Clinical Practice Phase	Control Group		Experimental Group		
		Learning activity of students	Teaching activity: Offline teaching	Learning activity of students	Teaching activity: Blended coaching	
					Online	Offline
Motivation	Pre-clinical	Perform preparation and pre-study	-Check preparation and assignment -Motivate practice	-Preparation and pre-study -Understanding and setting practice goals and strategies -Check clinical performance with structured questionnaire -Establish daily goal-setting during practice and prepare practice plan	-Check practice goals, encouraging pre-study before practice -Check preparation and assignment -Identify learning needs and motivate students with questions and discussions	-Explanation of self-directed learning strategies
Self-Management	Clinical	-Set daily goal -Implementation of nursing process regarding the assigned case in pediatric clinic -Performance of fundamental nursing skills and health assessment in children -Prepare daily practice report	-Teaching case/nursing processes in pediatric clinic -Teaching nursing skills and health assessment in children	-Implementation of nursing process regarding the assigned case in pediatric clinic -Performance of fundamental nursing skills and health assessment in children -Set daily goal -Prepare and post daily practice report and reflection report -Self-assessment and reflection on modifying daily goal setting and achieving daily goals	-Conduct Q/A frequently -Provide references -Encourage and provide feedback on daily practice and reflection reports -Check practice goal achievement -Discuss weaknesses and improvement points	-Teaching case/nursing processes in pediatric clinic -Teaching nursing skills and health assessment in children
Self-Monitoring and Evaluation	Post-clinical	-Analysis of case study -Self-report on practice (structured questionnaire) -Sharing practice impressions	-Analysis of case study	-Analysis of case study -Self-report about practice (structured questionnaire) -Self-reflection on overall clinical practice -Discuss practice-related impressions and improvements		-Feedback about analysis of case study -Discussion about clinical practice experiences

### Assessments

#### General characteristics

The included variables of the students were age, self-evaluation of previous semester academic achievement, and satisfaction in nursing.

#### Self-directed learning competency

We used the 45-item Self-Directed Learning Competency Questionnaire, which was developed for undergraduates/adults by Lee et al. [15] and includes three subscales: planning (20 items), implementation (15 items), and evaluation (15 items). Items are rated on a 5-point Likert scale (1 = almost never to 5 = almost always). The total score ranges from 45 to 225, with higher scores indicating higher levels of self-directed learning competency. The internal reliability coefficient in the Lee et al. [15] study was 0.93. In this study, the reliability coefficients were 0.89 (pretest) and 0.90 (posttest), and the subscale internal reliability ranged from 0.72 to 0.86.

#### Perceived clinical competency

Perceived clinical competency was measured with a questionnaire developed for nursing students by Lee et al. [16]. This is a 45-item, self-report instrument that assesses five subscales: nursing process (11 items), nursing skill (11 items), degree of cooperation (8 items), personal relations/communication (6 items), and professional development (9 items). Items are rated on a 5-point Likert scale (1 = almost never to 5 = almost always). A higher score indicates a greater degree of perceived clinical competency. The internal reliability coefficient in the original study was 0.96. In this study, the reliability coefficients were 0.96 (pretest) and 0.95 (posttest).

#### Clinical practice satisfaction

A numeric rating score (NRS) was used to measure clinical practice satisfaction. Participants were asked to evaluate their degree of satisfaction with the whole clinical practice, homework, content, and self-reflection on a scale of 0 to 10. A higher score indicates a greater degree of clinical practice satisfaction.

### Procedure

We used a non-synchronized design to prevent diffusion of treatment. In the control group that was trained using conventional self-directed learning program based on offline teaching, participants were recruited from September 1 to October 25, 2015, which were the beginning and end dates of the clinical practice period. In the experimental group that was trained using SDL_BC, participants were recruited from August 29 to October 28, 2016. Students were unaware whether they belonged to the control or experimental group.

Data were gathered in two phases, approximately 2 weeks apart. Participants completed self-report questionnaires. Pre- and post-intervention data were collected before and after the pediatric nursing clinical practice, respectively, and included demographic characteristics, self-directed learning, and clinical competency. The assessment of clinical practice satisfaction was performed immediately after completing the clinical practice. The questionnaires were completed in 15–20 min in both phases.

### Ethical considerations

Written informed consent was obtained from all participants before inclusion in the study, which was previously approved by the Sungshin Women’s University Institutional Review Board (SSWUIRB 2015–056, SSWUIRB 2016–022). As for the ethical aspects of the procedure, participants were informed of the purpose of the study and their right to refuse to participate at any time. Participants were informed that there would be no penalties for refusing to complete the survey. Researchers then distributed and collected the questionnaires onsite. Students used a self-chosen code to reconcile their pre- and post-intervention questionnaires, thus assuring data anonymity and confidentiality.

### Data analysis

Data were analyzed using the Statistical Package for the Social Sciences (SPSS), version 20 (SPSS Inc., Chicago, IL, USA). A Shapiro-Wilk test was performed to verify the normal distribution of the study variables, and parametric tests were subsequently used. Descriptive statistics, such as mean, standard deviation, frequency, and percentage, were calculated to assess the general characteristics of participants. The pre-intervention test of homogeneity was analyzed using an independent t-test and Fisher’s exact test. The pre-intervention test of homogeneity for self-directed learning and clinical competency between the experimental group and the control group was analyzed using the independent t-test. ANCOVA analyses that were controlled for pretest scores as confounders were performed to examine the effect on self-directed learning and clinical competency. An independent t-test was also used to examine the effects on the clinical practice satisfaction score in the experimental and control groups [17]. The significance level is based on *P* < .05.

## Results

### Participants

The general characteristics of the two groups are shown in Table 2. No significant differences in general characteristics were found between the control and experimental groups. In addition, baseline scores of the main variables (self-directed learning competency and perceived clinical competency) were not significantly different. Therefore, the two groups were considered homogenous.

**Table 2:** Homogeneity Test of General Characteristics and Pretest Competency Score (*n* = 91)

Variable		Control Group (*n* = 44)	Experimental Group (*n* = 47)	*X*^2^ or t	*P* value
		n (%) or M+/−SD	n (%) or M+/−SD		
Age^a^		21.48 ± 1.65	21.45 ± 1.63	−0.09	0.930
Academic achievement (previous semester)^b^	High	4 (9.1)	6 (12.8)	0.60	0.797
	Middle	33 (75.0)	32 (68.1)		
	Low	7 (15.9)	9 (19.1)		
Satisfaction in nursing^b^	Very satisfied	4 (9.0)	6 (12.8)	2.81	0.423
	Satisfied	16 (36.4)	20 (42.6)		
	Average	15 (34.1)	14 (29.8)		
	Dissatisfied	4 (9.0)	7 (14.9)		
Self-directed learning competency^a^		161.14 ± 14.99	163.09 ± 17.77	0.56	0.575
Perceived clinical competency^a^		156.43 ± 17.72	162.70 ± 23.98	1.41	0.162

^a^ independent t-test, ^b^ Fisher’s exact test

### Intervention effects

Table 3 presents the differences in self-directed learning competency and perceived clinical competency scores between the experimental and control groups. Compared with the control group, the pre-post difference in competency in implementation of self-directed learning in the experimental group was significantly greater (F (1,89) = 4.27, *p* = 0.039). There were no statistically significant differences in other variables related to self-directed learning competency. In the case of perceived clinical competency, there were no statistically significant differences between the two groups (t (89) = − 0.06, *p* = 0.956). On the other hand, regarding clinical practice satisfaction, there were significant differences in overall clinical practice (t (89) = 3.10, *p* = 0.003), practice content (t (89) = 3.88, *p* < 0.001), and self-reflection (t (89) = 3.36, *p* = 0.001) (Table 4). No significant difference was found for homework.

**Table 3:** Differences in Self-directed Learning and Perceived Clinical Competency Scores between Experimental and Control Groups (*n* = 91)

Independent variable and covariate	Cont.	Exp.	F	*P* value	95% CI	
	M+/−SD	M+/−SD			Lower	Upper
Self-directed learning competency (total)						
Pretest (covariate)	161.14 ± 14.99	163.09 ± 17.77	1.35	0.249	−2.11	8.04
Posttest	163.34 ± 17.42	167.94 ± 19.20				
Planning						
Pretest (covariate)	66.89 ± 7.48	70.66 ± 9.64	0.23	0.632	−2.19	3.60
Posttest	70.30 ± 8.61	73.51 ± 9.12				
Implementation						
Pretest (covariate)	57.32 ± 5.78	55.85 ± 6.31	4.40	0.039	0.11	4.27
Posttest	56.41 ± 6.62	57.38 ± 7.16				
Evaluation						
Pretest (covariate)	36.93 ± 4.55	36.57 ± 4.50	0.75	0.390	−0.86	2.19
Posttest	36.64 ± 4.96	37.04 ± 4.84				
Perceived clinical competency						
Pretest (covariate)	156.43 ± 17.72	162.70 ± 23.98	0.09	0.768	−5.11	6.89
Posttest	162.77 ± 21.88	168.87 ± 23.22

**Table 4:** Differences in Clinical Practice Satisfaction Scores between Experimental and Control Groups (*n* = 91)

Variable	Control	Experimental	Independent t-test	*P* value	95% CI	
	M+/−SD	M+/−SD			Lower	Upper
Whole clinical practice	7.10 ± 1.88	8.13 ± 1.17	3.10	0.003	0.38	1.67
Homework	7.34 ± 1.65	7.28 ± 1.85	−0.17	0.862	−0.79	0.67
Content	6.98 ± 1.99	8.34 ± 1.29	3.88	< 0.001	0.66	2.05
Self-reflection	7.23 ± 1.77	8.40 ± 1.57	3.36	0.001	0.48	1.87

## Discussion

The present pre-post non-synchronized intervention non-equivalent control group study evaluated a SDL_BC for undergraduate nursing students with a focus on self-directed learning competency, perceived clinical competency, and clinical practice satisfaction. Self-directed learning programs have been known to improve self-directed learning abilities in many previous studies [1, 5, 18]. In this study, blended coaching was applied to the self-directed learning program.

Findings from this study partially support our hypothesis: although the implementation of self-directed learning competency and clinical practice satisfaction increased significantly in the experimental group, we failed to observe significant differences between the control and experimental groups in competency in planning and evaluation of self-directed learning or perceived clinical competency.

The blended coaching provided to the experimental group in this study increased the online interaction relative to the control group, who received offline teaching. This online coaching included providing students with materials, answering questions, giving feedback on daily and reflection records, and encouraging discussion. These coaching methods improved students’ abilities to self-manage learning during the course. Immediate feedback is known to facilitate students’ motivation to learn and continuous learning management [19]. The students who participated in this study were able to ask questions during the clinical practice using mobile devices, and the coach provided immediate answers and a variety of materials as soon as possible. In fact, blended learning is less monotonous than face-to-face teaching and can enrich learning by providing various supplementary materials, as shown in this study [20].

In addition, one of the main focuses of this study was the online discussion process. Online discussion is considered more likely to be perceived as an easy and comfortable way to access educational materials, and can play a role not only in individual learning but also in communication and cooperative learning, which is considered to maximize the effectiveness of self-directed learning [21].

We could increase the amount of teaching time through blended coaching including the above methods. Blended learning not only overcomes constraints related to physical distance through the use of information and communication technology, but also reduces the transactional distance between instructor and learner, as well as between learners, through processes of teaching and learning that promote communication and cooperation [22]. Specifically, nursing students complete a challenging clinical practicum within a compressed time frame. Faculty members must make optimal use of teaching and learning time to equip students with a broad base of nursing knowledge and clinical reasoning skills through blended learning. Blended coaching can also complement the educational distance of face-to-face lectures.

As such, we used learning time and space, and interaction and learning methods in offline and online education flexibly. However, there was no significant difference between the experimental and control groups in planning and evaluation part. We believe that planning and evaluation parts provided through blended coaching did not differ from those of offline contents. We recommend developing a program emphasizing coaching and including various contents in planning and evaluation.

Perceived clinical competency was not significantly different between the two groups. We suggest that further programs need to combine other teaching and learning methods such as simulation practice or clinical examinations with blended coaching and test the effects on clinical performance [23, 24] to enhance students’ clinical competency.

In terms of clinical practice satisfaction, ratings of content and self-reflection were significantly higher in the experimental group, who received the blended coaching, except in the homework category. There are many studies showing that students are highly satisfied with the self-directed learning method [5, 25]. Students expressed satisfaction with the practice of self-directed learning based on blended coaching in the present study. We believe that the provision of diverse materials, discussions, and immediate feedback increased students’ clinical practice satisfaction. In addition, it appears that self-reflection during practice was made more effective by daily records of self-reflection, discussion of results, and feedback [26]. Additionally, nursing students were more satisfied with blended learning because of easy access to educational content. Blended learning can promote collaborative, learner-centered knowledge construction and offer a more comfortable environment.

In the homework category, the score was not significantly lower in the experimental group. This may be because the interaction loads between the coach and the student increase as the amount of learning increases. It is necessary to modify the program by giving students only pre-planned homework, and creating an environment in which the burden of extra homework is not unexpectedly increased and interesting learning is possible.

### Limitation

This study had several limitations. First, the participants were recruited from a single university in Korea, possibly limiting the generalizability of our findings. Second, the sample size was smaller than what was necessary according to the calculations using G*power. Third, the time between observations was 2 weeks, which is not a long-term follow-up to support our hypothesis. Forth, clinical competency was assessed by student themselves only, and we could not compare professor ratings and students’ scores. Finally, some variables were not measured, such as personality and learning style, which might affect the learning outcomes.

## Conclusion

We suggest that the present SDL_BC, involving blended learning (combining with online and offline methods) and coaching for clinical practice, was appropriately applied to self-directed learning among nursing students. The SDL_BC developed in this study was a suitable educational approach to improve the implementation of self-directed learning competency and clinical practice satisfaction. This seems to be a useful method for educators as they face the challenge of applying a variety of learning methods to enhance the competency of a new generation of students. Further research is required to investigate whether other variables are associated with clinical competency, competency in planning and evaluation of self-directed learning, and their long-term effects.

## Data Availability

The datasets generated and/or analysed during the current study are not publicly available due due to IRB regulation of our institution related to personal information but are available from the corresponding author on reasonable request.

## Abbreviations

ANCOVA: Analysis of covariance
NRS: Numeric rating score
SDL_BC: Self-directed learning program using blended coaching
SPSS: Statistical package for the social sciences
